# Event and Comment

**Published:** 1919-04

**Authors:** 


### DENTAL REGISTER

THE MAGAZINE OF DENTISTRY

Vol. LXXIII APRIL, 1919 No. 4

### EVENT AND COMMENT

**The Sears Impression and Bite Methods**

We are reprinting in this issue from the *Dental Digest* a method of impression taking which utilizes the combined modeling compound and plaster of Paris materials to excellent advantage in securing the best qualities of both materials. It also is a combination of the modeling compound methods of Green and Supplee with the Rupert Hall plaster methods, and is a step nearer perfection in impression taking than either of these methods. For those who can not get away from modeling compound as an impression material, we believe it has decided advantages, particularly in securing accuracy of form in the average mouth and certainly will give all the detail required in most cases. As a method for the usual case it is certainly worthy of careful consideration, and we commend it to our readers. The article in full, with illustrations, appears in the December, 1918, and January, 1919, *Dental Digest.*

The criticism we would make of the method is that while it in a considerable degree is capable of securing accuracy in form and detail of the mouth surface, it is lacking in the one important quality which seems to be overlooked by all prosthetic workers, namely, the restoration of the soft tissues to a normal condition. This we realize is not especially indicated in many cases, as the tissues do not always require therapeutic interference. But in most cases where a denture has been worn for some time, and which has not been perfectly adapted or kept in a hygienic condition, the soft structures are liable to be excessive and often pathologic. Any effort to adapt new dentures to such conditions which does not specifically plan to do all that is practicable to overcome such conditions, falls far short of accomplishing all that such cases require. It is true that this can be done by refitting such cases or by a new denture that supports the relaxed tissues, or by placing in contact with these tissues such plate materials as will exert a healing influence, but we are confident that much more can be done by using impression materials that will secure the maximum of physical restoration of these relaxed or redundant tissues to their normal form. Many operators try to accomplish this by carving the models so as to throw extra pressure on the soft areas, but this is indefinite and very difficult to accomplish, and few dentists will take the time to study the mouth sufficiently to enable them to do this accurately, nor can it be well done by compound.

There is one impression material, and only one, in our estimation, that can be depended upon to do this definitely and expeditiously, beeswax is the only one with which anyone who will master its technic can secure adequate results with the desired certainty. Plaster of Paris and modeling compound are both useful materials, and when used as Dr. Sears and Dr. Hall advocate, they will give excellent results. But beeswax excells in securing what we call a differential or discriminating impression. It may be said that the same discrimination can be secured by manipulating the other materials with this idea in mind. It is possible to so manipulate plaster or compound as to secure this ideal in a measure, but because of the inherent adhesive qualities of wax and the practicability of manipulating it through a longer range of varying degrees of plasticity, it is an ideal material for reducing the tissues to a more uniform condition of equable resistance than is possible with the other materials; and what is more, it can be done by the average technician.

If the objection be made that wax will not give the desired surface detail, this can easily be overcome by using the wax impression after it has been properly trimmed of excess as a tray for taking a plaster impression, which will give perfection of detail without losing any of the desired tissue correction by the wax.

This is a most timely matter and the profession ought to restudy this problem, not only from its technical bearings, but from the more important viewpoint of oral hygiene.

